# An Update on Blood Pressure Short-Term Variability

**DOI:** 10.1100/tsw.2002.102

**Published:** 2002-02-05

**Authors:** Nina Japundžić-Žigon

**Affiliations:** Institute of Clinical Pharmacology, Pharmacology, and Toxicology, Medical Faculty, University of Belgrade, Yu-11000 Belgrade, Serbia

**Keywords:** blood pressure variability, spectral analysis

## Abstract

This article summarizes current achievements of computer-based methodologies in identifying the constituting components of short-term blood pressure variability and the underlying regulatory mechanisms. It emphasizes the contribution of spectral methodologies in early diagnosis of blood pressure deregulations. Moreover, the effects of antihypertensive drugs, as well as of new, nonpeptide vasopressin antagonists on short-term blood pressure variability, are briefly described.

In the last decade, the development of computer-based analysis of cardiovascular (CV) signals allowed application of mathematical algorithms for the analysis of CV variability, and, for the first time, identification and quantification of the components of CV variability became available. Continuous recording of blood pressure (BP) and heart rate (HR) reveals that they oscillate in a complex manner: both periodic and nonperiodic components may be observed. In order to identify the constituting components of CV variabilities and study the underlying mechanisms, several methodologies were applied[1,2,3,4,5]. The periodic (oscillatory) components of CV variability were approached by spectral methods based on fast Fourier transform or autocorrelation[1,2,3] while the nonperiodic components of HR variability were assessed by nonlinear statistical methods and fractal analysis[4,5]. By applying these methods to the analysis of CV variability in disease, numerous clinical studies suggest that they may have important prognostic value for identifying CV patients at high risk[2,5].

In most species, including man, the oscillatory components of BP variability were identified around three frequency regions: very low frequency (VLF), low frequency (LF), and high frequency (HF) region. The origin of these oscillations was found to differ, and even to involve more than one regulatory mechanism in their modulation. For instance, VLF and LF oscillations, which lie closely in the BP spectrum, were associated with changes in the diameter of small, resistance arteries (peripheral resistance) located in different arterial beds[6]. Under basal conditions, the VLF oscillation represents most of the spectral power. Thermoregulation, the renin-angiotensin system (RAS), inherent myogenic activity of blood vessels (myogenic oscillations ≈0.13 Hz in rats), and autoregulatory mechanisms of local blood flow have been implicated in its genesis, whereas the baroreceptor reflex (BRR) was found to buffer them[6,7,8,9]. In contrast, LF oscillations (also known as neurogenic oscillations and Mayer waves) appear clearly in the BP spectrum only under stressful conditions and reflect increased sympathetic outflow to resistance vessels[3,10,11]. A "peaky" spectral presentation of LF oscillations (at ≈0.4 Hz in rats and ≈0.1 Hz in humans) indicates that a positive feedback control of the arterial baroreceptor reflex (resonance phenomenon) has occurred[11]. The HF oscillation is the smallest oscillation in the BP spectrum and it is often referred to as the "mechanical" oscillation. It reflects a small decrease of BP associated with inspiration due to the distension of the chest that affects circulation in thoracic vessels and the heart[3].

Changes in BP and HR variability associated with cardiovascular disease have been identified both in experimental models and humans[12,13,14,15]. In subjects suffering from peripheral autonomic neuropathy, the BP variability was found to be reduced, especially the neurogenic components[12,13]. On the other hand, borderline hypertensive subjects were found to exhibit increased LF variability to stress (orthostatic, mental, etc.) most probably as a consequence of excessive sympathetic outflow to the periphery[14]. However, the LF increase seems to be an inconsistent finding, and it has not been detected in established hypertension, in which BP spectral power is shifted to the slower frequency range and associated with the nonfunctioning BRR[14,15]. Moreover, the increased BP variability in the VLF range and even slower frequencies was found to be associated with increased CV morbidity and mortality[16]. Thus, elucidating the mechanisms that underlie BP variability is of great interest, as BP regulation aimed at reducing BP variability may play an important role in reducing the risk of cardiovascular complications.

Experimental findings indicate that most antihypertensive drugs that reduce peripheral sympathetic outflow or sympathetic transmission and reduce vascular resistance—such as centrally acting imidazoline receptor agonists, peripherally acting $\alpha$ adrenergic antagonists, RAS inhibitors, angiotensin II antagonists, and arterial vasodilators—decrease BP variability in the lower frequency region[3,6,7,8,17]. However, strong vasodilators, selective $\alpha_1$ adrenergic antagonists, or ganglionic blocking agents tend to increase the HF oscillation by unloading thoracic veins[3]. Furthermore, strong diuretics in doses that produce normotensive hypovolaemia recruit vasoconstrictor mechanisms (sympathicus, RAS, and vasopressin) which in turn, enhance BP variability in all frequency regions, including the VLF and LF regions[18]. We have recently reported experimental evidence about the effects of new nonpeptide and selective vasopressin $V_{1a}$ and $V_2$ antagonists on BP variability[19]. Our results in normotensive rats show that vasopressin antagonists enhance VLF BP variability, suggesting that vasopressin contributes to the buffering of VLF BP variability. Preliminary results in genetically hypertensive SH rats further indicate that the buffering effect of vasopressin on VLF oscillations is impaired. Thus, we believe that vasopressin $V_{1a}$ antagonists, in addition to being poor hypotensive agents, do not seem to be promising drug candidates for monotherapy in hypertension.

The nonperiodic component of BP variability appears in the BP spectrum in all frequency domains as a widespread "noise." Its source is still unknown and its contribution to diagnosis of cardiovascular disease remains to be established. Experiments on isolated blood vessel preparations suggest that vascular smooth muscles of resistance vessels have inherent nonperiodic dynamics created by multiple membrane and intracellular $Ca^{2+}$ fluxes[20]. They also provide evidence that the level of intra-arterial pressure may determine a degree of nonlinear dynamics. Nonetheless, studies on HR variability indicate that the pattern of nonperiodic HR variability is not entirely unpredictable and that it has a fractal structure[4,5]. They also indicate that the loss of nonlinearity may anticipate serious deterioration of cardiac disease. Therefore, nonlinearity in CV signals seems to play an important role in ensuing flexibility and efficiency of CV control. Research aimed at elucidating the mechanisms that underlie CV variability will contribute to better understanding of the CV control mechanism and the pathophysiological changes in CV disease, and will promote a critical reassessment of CV therapy.

## BIOSKETCH

**Nina Japundžić-Žigon,** M.D., Ph.D., is Associate Professor of Pharmacology and Toxicology, Faculty of Medicine, Belgrade University. She earned her Ph.D. (Pharmacology), M.Sc. (Endocrinology), and M.D. (magna cum laude) degrees from the University of Belgrade. Her research interests include autonomic and humoral control of arterial blood pressure and heart rate short-term variability; computer analysis of cardiovascular signals in time and frequency domain. Currently Dr. Japundžić-Žigon is working on the effects of vasopressinergic antagonists on blood pressure short-term variability in conscious normotensive and hypertensive models of rats (SHR, DOCA-salt), and the effects of antiarrhythmic drugs on the heart rate variability in children. She has been the recipient of many awards and honors, including the Bourse de l' Association Claude Bernard for visiting scientists (1999-2000), Young Investigator Award of ICN Pharmaceuticals Yugoslavia (1995), Postdoctoral scholarship from Association Claude Bernard (1990), Stagiaire du Gouvernement Français (1988-1989), National Scholarship for the highly gifted (1986-1988), and "Award of the town of Belgrade" for the best Student's Scientific Paper (1986).
